# Using wearable biological sensors to provide personalized feedback to motivate behavioral changes: Study protocol for a randomized controlled physical activity intervention in cancer survivors (Project KNOWN)

**DOI:** 10.1371/journal.pone.0274492

**Published:** 2022-09-13

**Authors:** Yue Liao, Susan M. Schembre, Grace E. Brannon, Zui Pan, Jing Wang, Sadia Ali, M. Shaalan Beg, Karen M. Basen-Engquist

**Affiliations:** 1 Department of Kinesiology, College of Nursing and Health Innovation, The University of Texas at Arlington, Arlington, Texas, United States of America; 2 Department of Oncology, Georgetown Lombardi Comprehensive Cancer Center, Georgetown University, Washington, DC, United States of America; 3 Department of Communication, College of Liberal Arts, The University of Texas at Arlington, Arlington, Texas, United States of America; 4 Department of Graduate Nursing, College of Nursing and Health Innovation, The University of Texas at Arlington, Arlington, Texas, United States of America; 5 Department of Internal Medicine, UT Southwestern Medical Center, Dallas, Texas, United States of America; 6 Department of Health Disparities Research, The University of Texas MD Anderson Cancer Center, Houston, Texas, United States of America; Public Library of Science, UNITED KINGDOM

## Abstract

Regular physical activity reduces the progression of several cancers and offers physical and mental health benefits for cancer survivors. However, many cancer survivors are not sufficiently active to achieve these health benefits. Possible biological mechanisms through which physical activity could affect cancer progression include reduced systemic inflammation and positive changes in metabolic markers. Chronic and acute hyperglycemia could have downstream effects on cell proliferation and tumorigenesis. One novel strategy to motivate cancer survivors to be more active is to provide personalized biological-based feedback that demonstrates the immediate positive impact of physical activity. Continuous glucose monitors (CGMs) have been used to demonstrate the acute beneficial effects of physical activity on insulin sensitivity and glucose metabolisms in controlled lab settings. Using personal data from CGMs to illustrate the immediate impact of physical activity on glucose patterns could be particularly relevant for cancer survivors because they are at a higher risk for developing type 2 diabetes (T2D). As a pilot project, this study aims to (1) test the preliminary effect of a remotely delivered physical activity intervention that incorporates personalized biological-based feedback on daily physical activity levels, and (2) explore the association between daily glucose patterns and cancer-related insulin pathway and inflammatory biomarkers in cancer survivors who are at high risk for T2D. We will recruit 50 insufficiently active, post-treatment cancer survivors who are at elevated risk for T2D. Participants will be randomly assigned into (1) a group that receives personalized biological feedback related to physical activity behaviors; and (2) a control group that receives standard educational material. The feasibility and preliminary efficacy of this wearable sensor-based, biofeedback-enhanced 12-week physical activity intervention will be evaluated. Data from this study will support the further refinement and enhancement of a more comprehensive remotely delivered physical activity intervention that targets cancer survivors.

**Trial registration:** ClinicalTrials.gov Identifier: NCT05490641.

## Introduction

Considerable epidemiologic research suggests that physical activity can reduce cancer-related and overall mortality in survivors of several cancers [1, 2]. For example, physical activity has 28–38% risk reduction in cancer-specific mortality for breast cancer and 39% risk reduction for colorectal cancer [3]. Randomized clinical trials have also provided strong evidence that physical activity after cancer diagnosis offers significant improvement in survivors’ physical outcomes (e.g., increased fitness and physical functioning, decreased fatigue) and mental health (e.g., increased self-esteem, decreased depression and anxiety) [4–8]. This scientific evidence of the numerous benefits of physical activity has resulted in an updated exercise guideline for cancer survivors in 2019, which calls for every survivor to engage in adequate physical activity [9].

Possible biological mechanisms through which physical activity could affect cancer progression include decreased levels of circulating sex hormones (e.g., estrogens, androgens), reduced systemic inflammation, and positive changes in metabolic markers (e.g., decreased insulin and glucose levels) [10]. Chronic and acute hyperglycemia could have downstream effects on cell proliferation and tumorigenesis, possibly via modulation of the insulin-like growth factor (IGF) axis [11]. Previous physical activity interventions showed beneficial changes in biomarkers implicated in these pathways, including insulin, leptin, IGFs, and C-reactive protein (CRP) in cancer survivors [12–14]. For example, after a 15-week supervised aerobic exercise intervention, a significant decrease in IGF-1 and CRP was observed in postmenopausal breast cancer survivors [15, 16]. A significant reduction in leptin was also observed after a 12-week physical activity intervention in breast cancer survivors [17]. In overweight and obese adults, a significant decrease in inflammatory biomarkers such as interleukin-6 (IL-6) and tumor necrosis factor alpha (TNF-α) was found after a 12-week aerobic exercise intervention [18] and a 16-week low-intensity, internet-delivered physical activity intervention [19]. Nevertheless, despite the evidence of the beneficial effects of physical activity, approximately 84% of cancer survivors are not sufficiently active in their daily lives [20, 21]. Therefore, to improve post-treatment cancer outcomes and quality of life for cancer survivors, it is vital to promote an active lifestyle for this population through effective behavioral interventions [22, 23].

One of the key behavioral change strategies in physical activity intervention is the delivery of performance feedback [24–26]. Several behavioral change theories advocate the use of performance feedback, postulating that feedback on current performance relative to behavioral goals motivates behavioral change [27–29]. However, the effects of performance feedback on behavioral changes are inconsistent [30, 31]. One reason for this inconsistency could be that performance-based feedback alone may not be sufficiently motivating. In particular, physical activity is often characterized by the expense of immediate effort without a tangible short-term benefit [32]. Thus, to enhance the effectiveness of providing feedback to motivate physical activity, there is a critical need to develop methods that could help individuals grasp the health benefits of physical activity in a more immediate and concrete way.

Providing feedback on individuals’ biological indices has been used to increase motivation and promote behavior change in the past [33]. In fact, biofeedback is one of the behavioral change techniques under the category of feedback and monitoring [34]. However, biofeedback has not been fully utilized in physical activity interventions. Examples of existing biofeedback used in physical activity interventions include review blood pressure and weight at baseline and follow-up assessment [35]. Given the rapid advancement in wearable sensor technologies that has made continuous monitoring of personal biological data more accessible, we see a great opportunity to further develop and apply personalized biofeedback in physical activity interventions for cancer survivors. Specifically, we could incorporate performance-based feedback typically provided in a physical activity intervention into a biological outcome that is favorably and acutely affected by physical activity to increase motivation for behavioral changes. For such feedback to be personally relevant for cancer survivors, the candidate biological outcome must have long-term clinical implications for cancer survivorship and disease outcome. Therefore, we propose to use glucose data as the basis for providing such biofeedback in cancer survivors to motivate physical activity for two main reasons: First, acute bouts of physical activity can improve insulin sensitivity and increase glucose uptake by skeletal muscles [10] (i.e., the immediacy of the physical activity effects). Second, cancer survivors are at a high risk of developing type 2 diabetes (T2D) compared to those without cancer diagnosis [36–38], and elevated blood glucose in nondiabetic cancer survivors has been associated with poor prognosis [39, 40] (i.e., the relevance of glucose data to cancer survivors). In cancer survivors, T2D is one of the most common comorbid conditions. T2D or impaired glucose tolerance has been found to be associated with increased cancer mortality, especially in the physically inactive population [10].

In recent years, an increasing number of studies have used continuous glucose monitors (CGMs), which measure glucose concentrations in the interstitial fluid in real-time through a tiny sensor inserted under the skin, to obtain more frequent readings (e.g., every 5–15 minutes) of glucose data to better illustrate this acute impact of physical activity on insulin sensitivity and glucose metabolism in controlled laboratory settings [41–49]. Few studies, however, have used CGMs outside of laboratory settings to provide feedback on the immediate benefits of physical activity on daily glucose patterns. Preliminary data from our research group show that physical activity interventions featuring the use of CGMs are highly feasible and acceptable in sedentary overweight and obese adults [50]. Previous qualitative studies with breast and colorectal cancer survivors also indicate high acceptability of CGM-based biofeedback to promote physical activity [51]. Taking together, utilizing data from CGM to provide personalized biofeedback is highly promising in relaying the immediate benefits of physical activity and the long-term health outcomes for insufficiently active cancer survivors who are at high risk for T2D. This line of research (i.e., leveraging biosensor data to deliver personalized and timely feedback messages to motivate physical activity) has great potentials in scaling up in the future as there are already several startup companies offering CGM directly to consumers for personalized nutrition purpose (despite lack of incorporating evidence-based behavioral change strategies) [52].

Further, previous studies have found that physical activity and chronic hyperglycemia (i.e., elevated hemoglobin A1c) are correlated with certain cancer-related biomarkers (e.g., insulin-related pathways and inflammation) [11, 53–55]. However, little is known about whether daily glucose patterns (e.g., 24-h average, glucose variability, acute hyperglycemia) are associated with these cancer-related biomarkers in cancer survivors. Thus, identifying daily glucose patterns that might serve as mediators of the association between physical activity and changes in cancer-related biomarkers will lead to practical implications for cancer survivorship care and program design.

The objectives for this pilot project are: (1) test the preliminary effect of a remotely delivered physical activity intervention that incorporates personalized biological-based feedback on daily physical activity levels, and (2) explore the association between daily glucose patterns and cancer-related insulin pathway and inflammatory biomarkers in cancer survivors who are at high risk for T2D.

## Methods

### Participants recruitment and eligibility

We will recruit post-treatment cancer survivors who live in the Dallas-Fort Worth metroplex area using several recruitment strategies. We will utilize the Commission on Cancer (CoC) accredited Tumor Registry for the University of Texas Southwestern (UTSW) Simmons Comprehensive Cancer Center and the Dallas County Safety Net Hospital, Parkland Health and Hospital Services to identify potentially eligible patients. We will also work with clinicians at the UTSW Cancer Center and Parkland Health to help us identify potentially eligible participants in their clinics. To further expand our patient pool to include a diverse patient population, additional patients could be recruited from local communities through social media, research study recruitment website (e.g., Research Match), tabling at survivorship events, and posting notifications in newsletters and Facebook pages of survivorship organizations. The study team has successfully recruited a diverse cancer survivor population for previous studies using these established recruitment methods.

Cancer survivors who express interest in the study will complete a brief screening questionnaire online or over the phone to determine eligibility. To be eligible for the study, individuals must be 18 years or older, have had a diagnosis of cancer; have completed curative-intended treatment for at least 3 months (except hormone therapy or long-term maintenance chemotherapy); be at high-risk for T2D based on the American Diabetes Association Type 2 Diabetes Risk Test [56, 57] (a score of 5 or higher based on the seven screening questions), be insufficiently active (engaging in < 90 minutes of moderate-intensity aerobic physical activity per week); have no contraindications to exercise (either no positive responses on the Physical Activity Readiness Questionnaire [58], or clearance from a health care provider certifying that the patient is healthy enough to exercise); have no current diagnosis or history of type 1 or 2 diabetes; not on a low-carb diet; and have a smartphone with daily internet access. Those who are eligible based on these criteria will be scheduled for an in-person appointment and be asked to consent for the randomized study. Reasons for ineligibility and refusing study enrollment will be documented.

### Study procedures

**Fig 1:**
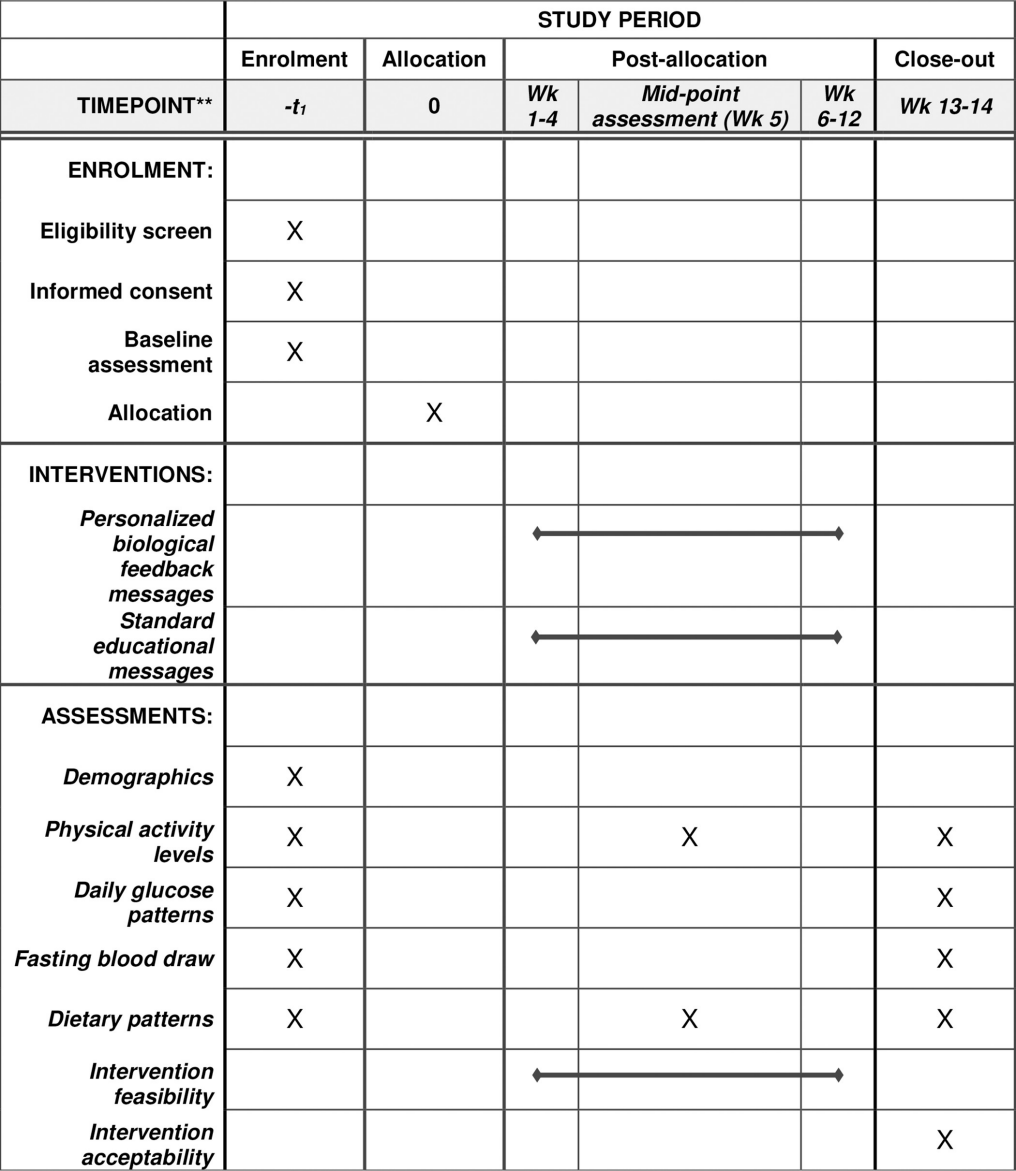
Schedule of enrolment, interventions, and assessments.

Eligible participants will be randomly assigned to a 12-week physical activity intervention that features personalized biofeedback based on their CGM data or a control group. All participants will start with a baseline assessment, including questionnaires, fasting blood draw, and 2-week monitoring with an accelerometer and a blinded CGM. During the intervention period, participants in both groups will wear a Fitbit device throughout the 12-week period. The control group will receive standard educational text messages 2–3 times per week. The intervention group will additionally wear an unblinded CGM and receive personalized biofeedback messages in the first 4 weeks of the intervention period. A mid-intervention assessment with accelerometer monitoring and dietary assessment will occur in week 5. All participants will return for a post-intervention assessment, which will include questionnaires, exit interview, fasting blood draw, and 2-week monitoring with an accelerometer and a blinded CGM. Participants will be able to keep the Fitbit device and be compensated (up to $50) for completing this study. Fig 1 shows the schedule of enrollment, interventions, and assessments. Our study protocol followed the SPIRIT (Standard Protocol Items: Recommendations for Interventional Trials) guideline (see appendix) [59]. The first version of this study protocol was approved by the University of Texas at Arlington’s Institutional Review Board (protocol #: 2022–0177) on June 8, 2022. Continuing review will be performed by the IRB annually. Any serious adverse events and unanticipated problems will be reported to this institutional IRB. Amendments to the protocol will be reviewed and approved by the IRB before implementation. This study is registered on ClinicalTrials.gov (NCT05490641). Because this is a pilot study that involves minimal risks, a data monitoring committee is not needed.

#### Baseline assessment

Eligible participants will be scheduled for an in-person visit at the Physical Activity and Wearable Sensors (PAWS) Research Laboratory, located at the UT Arlington campus in Arlington, Texas. At this in-person visit, participants will be asked to provide written consent for enrollment in the study. The baseline assessment consists of five major components which include in-person procedures in the lab and a 2-week free-living monitoring. **(1)** Participants will have their height and weight measured by study staff, and will complete a set of questionnaires assessing demographics (e.g., age, gender, race and ethnicity, marital status, education level, household income, employment status), cancer-related medical history (e.g., cancer type and stage, date of diagnosis, treatment type and history, medication use), quality of life [60], health literacy [61, 62], health information technology use, and physical activity-related psychosocial variables such as self-determination motivation [63], stages of change [64], and outcome expectancy [65, 66]. **(2)** Participants will complete a validated, self-administered, web-based graphical food frequency questionnaire, VioScreen [67]. The food frequency questionnaire will assess participants’ usual dietary intake in the past month. **(3)** Participants will have their fasting blood drawn by a certified phlebotomist. Collected blood plasma will be analyzed for relevant biomarkers including HbA1c, insulin, IGF-1, IGF-2, CRP, leptin, IL-6, and TNF-α. All blood samples will be deidentified and personnel who are involved in the blood sample analysis process will be blinded from participants’ group assignment. **(4)** Participants will be given a triaxial accelerometer (ActiGraph GT3X) for daily activity monitoring. This is a blinded device so that participants will not be able to view any of their activity data during this baseline assessment period. To minimize device non-wear and missing data, participants will be instructed to wear the accelerometer device (with a provided woven nylon wristband) on their non-dominant wrist at all times (including when sleeping and showering). The ActiGraph GT3X device is water resistant, and when fully charged, it can continuously record and store activity data at the minute-level for up to 25 days. Thus, there will be no need for participants to take off the device for charging purpose during the 2-week assessment period. **(5)** All participants will have a FreeStyle Libre Pro CGM sensor (Abbott Laboratories, California) inserted on the back of their upper arm. This CGM system is FDA-approved and comprised of a sensor and a reader. The CGM sensor is waterproof, weights 5 grams and measures at 5 mm height and 35 mm diameter. Once inserted and activated, the sensor will start recording interstitial glucose data every 15 minutes continuously for 14 days without the need for finger-stick calibration. The CGM sensor is designed to be self-inserted. Instructional videos provided by the manufacturer will be used to guide sensor insertion. If preferred, the sensor can be inserted by trained study staff. To blind the glucose data from participants, the reader will not be provided to them. The CGM sensor is waterproof. Therefore, once inserted, there will be no need for participants to remove the sensor. We will apply extra adhesives over the sensor to help secure its position and ensure the continuous collection of glucose data. Data are stored in the sensor and will be downloaded by study staff when the assessment period is completed. Participants will be told not to change their usual behaviors during the baseline free-living monitoring period.

#### Randomization and intervention visit

Participants will come back to UT Arlington campus for their intervention visit after the baseline free-living monitoring period. Prior to this intervention visit, participants will be randomized into either an intervention or a control group. We will use the minimization method, which randomizes participants based on the assignment that would provide the best overall balance with respect to selected covariates [68, 69]. Before a participant is assigned to a group, the number of participants in each group with similar covariate characteristics is totaled. These totals are based on marginal sums of the covariates so that each covariate is considered separately. Participants’ assignments are determined based on which group assignment provides the best overall balance with respect to covariates. We will use cancer type, gender, age, race/ethnicity, and weight category as covariates. We will be able to get this information from participants’ baseline visit to perform randomization. Intervention allocation will be sequentially numbered. Participants will be informed of their group assignment at their intervention visit. At the intervention visit, participants will return the baseline assessment equipment (i.e., accelerometer and CGM sensor) and will be given a Fitbit Inspire 2 fitness tracker to wear during the 12-week intervention period. Participants will be instructed to wear this Fitbit wristband at all times, including during sleeping. The Fitbit Inspire 2 is swim-proof and water-resistant to 50 meters. A fully charged Inspire 2 can continuously track activities and heart rate for up to 10 days. We will encourage participants to charge the Fitbit device, as needed, when they take a shower. A Fitbit mobile application (app), available in both Android OS and iOS, will be downloaded to participants’ phones. Study staff will teach participants how to keep the Fitbit device synced with the Fitbit app. Study staff will be able to monitor participants’ Fitbit information (including activity data, battery status, and syncing events) in real-time through Fitabase (Small Steps Labs LLC, California), a web-based platform that processes Fitbit data and generates activity reports and graphs. Reminders will be sent out if noncompliance (e.g., device non-wear, low battery, outdated syncing) is detected. Participants in the control group will receive standard educational materials about physical activity, while participants in the intervention group will receive the biofeedback-enhanced materials. Details about the intervention are described in the section below.

#### Mid-intervention free-living monitoring assessment

At the end of intervention week 4, we will mail all study participants the ActiGraph GT3X device for them to wear during week 5 (i.e., 7-day monitoring). All participants will also complete another food frequency questionnaire using VioScreen during week 5.

#### Post-intervention visit

Participants will return for a final assessment after the 12-week intervention period. They will complete the post-intervention questionnaires, study evaluation, exit interview, and provide their fasting blood samples. Participants will wear the blinded accelerometer and CGM for the next 2 weeks. They will also complete another food frequency questionnaire using VioScreen during this 2-week period. Participants will be able to keep their Fitbit device after completion of this study and receive up to $50 compliance-based compensation.

### Intervention

All participants will receive an educational handout that discusses prevalent comorbidities in cancer survivors highlighting the T2D risk, the short-term and long-term benefits of physical activity on cancer survivorship, and tips about becoming more active in their daily life. All participants will wear the Fitbit Inspire 2 wristband during the 12-week intervention period. Participants can track their daily steps, exercise minutes and intensity, as well as daily activity trends and progress towards goals through the Fitbit app. Participants will be encouraged to engage in at least 150 minutes of moderate-intensity aerobic physical activity each week. All participants will receive theory-based (e.g., social cognitive theory and self-determination theory) educational text messages 2–3 times per week that reflect the topics in the educational handout (e.g., benefits of physical activity for cancer survivorship, tips for exercising, reminders about goals). These messages are adapted from a previous intervention where a message bank was developed based on the Diabetes Prevention Program curriculum [70] and have been pilot-tested in the target population by the research team.

Participants in the intervention group will additionally wear an unblinded, personal FreeStyle Libre CGM during the first four weeks of the intervention period. This CGM system is similar to the FreeStyle Libre Pro, except that participants will be able to view their glucose information in real-time through an accompanying smartphone app. Study staff will help participants download the CGM app, available in both Android OS and iOS, and teach them how to use their phone to assess their glucose information. The smartphone app will display current glucose reading, a trend arrow indicates the direction the glucose is moving, and a graph shows an 8-hour history of the glucose value. These glucose data will be synced to a server wirelessly through the app. Study staff will be able to access participants’ CGM data and daily glucose pattern summary statistics remotely via LibreView, a web-based platform developed by the manufacturer to view participants’ CGM data and generate reports. A FreeStyle Libre sensor will continuously record interstitial glucose data for 14 days without the need for finger-stick calibration. Participants will need to replace the CGM sensor once for a total of 4-week monitoring. During this 4-week CGM monitoring period, participants will receive personalized biofeedback messages 1–2 times per week based on their Fitbit and CGM data. These biofeedback messages have been developed based on results from the focus group study in the target population (i.e., insufficiently active overweight/obese cancer survivors) [51]. The biofeedback messages incorporate participants’ Fitbit and CGM data with behavioral change theory-based topics such as goal-setting, self-monitoring, and outcome expectations. These biofeedback messages have been pilot-tested in the target population. Data to tailor these messages will be available from the LibreView platform and the Fitabase platform. During the weeks that participants are not wearing the CGM sensor, they will receive messages reminding them of the acute impact of physical activity on their glucose patterns. The 4-week CGM monitoring period with personalized biofeedback is meant to be an experiential learning experience for participants to link their daily behaviors with an immediate biological outcome. Through continuous activity monitoring via Fitbit during the 12-week intervention period, we will be able to examine the longitudinal changes in daily activity levels. If we detect an initial increase in activity levels in the first four weeks but then a substantial drop in activity levels in the intervention group in the following weeks, this will suggest a need for a "booster" biofeedback intervention dosage. With data from this pilot study, we will be able to determine the timing and duration of such "booster" intervention dosage for future trials.

### Statistical methods

#### Data analysis plan

Feasibility of providing personalized biofeedback will be assessed based on intervention adherence and retention. Intervention adherence for an individual is defined as wearing the Fitbit device (≥10 hr non-sleep wear time per day) for at least 70% of the intervention period and having non-missing CGM data for at least 70% of the CGM monitoring period. The intervention adherence at the study level is then defined by the percentage of participants adhering to the above-mentioned criteria. Retention will be assessed by the percentage of participants completing the final study assessments. We will calculate rates, frequencies, and 95% credible intervals (CrIs) for these measures. Criteria for feasibility will be defined as: i) study intervention adherence rate of ≥80%; and ii) a retention rate of ≥ 80%. Preliminary effects of the intervention will be evaluated on the following outcome variables at post-intervention: (1) daily average moderate-to-vigorous physical activity minutes; and (2) daily average sedentary minutes. Both variables will be measured by accelerometers using established cut-points for wrist-worn devices [71]. The intervention effect will be evaluated by performing the following analyses on the outcome variables: (1) calculating descriptive statistics (e.g., means, SDs, and 95% confidence intervals) by study group and group differences in outcome means; (2) performing a two-sample t-test for the group differences in outcome means; and (3) examining the between-group difference using linear regression analysis while controlling for potential confounders such as baseline outcome measurements and dietary intake. Additional exploratory statistical analyses will include the generalized linear mixed model to examine the longitudinal trajectories of daily activity levels over time using the continuous Fitbit data. All tests will use a two-sided 5% significance level. We will calculate metrics to identify daily glucose patterns (e.g., 24-hr average, daily variability, frequency of hyperglycemic events, time in hyperglycemic zone) using the blinded CGM data from baseline and post-intervention assessment. Simple linear regression will be used to examine the cross-sectional associations between daily glucose patterns and the biomarkers. Linear mixed model will be used to examine the changes in daily glucose patterns and biomarkers over time. We will further explore daily glucose patterns as a mediator of the relationship between changes in physical activity and biomarkers over time. These analyses will focus on obtaining preliminary information regarding associations between daily glucose patterns and biomarkers and thus will be exploratory in nature.

#### Sample size justification

Conducting future, similar trials would not be considered feasible with adherence and retention rates of 70%; these rates would need to be at least 80% to consider future trials’ feasibility. Therefore, we will consider future studies feasible if Pr[π_adhere_ ≥ 0.7 | data] > 0.8 and if Pr[π_retention_ ≥ 0.7 | data] > 0.8. Using results from our previous CGM pilot studies, we assume that both π_adhere_ and π_retention_ follow a beta(8, 2) distribution, which has a mean of 0.8 and a variance of 0.01. If π_adhere_ is truly 0.8, given the above rule, we have an 89% chance of concluding adherence is feasible. However, if it is only 0.7, with the above rule, we have a 33% chance of concluding that adherence is feasible. This also applies to the rule for declaring feasibility with regard to retention. Furthermore, assuming independence, we have a 79% chance of declaring the trial feasible. These endpoints are most likely positively correlated, so the probability of declaring the trial feasible is much likely higher under these rules. Assuming 80% retention in each arm and a standard deviation of 1, we will have 80% power to detect a 0.9 difference in means between arms using a 2-sided test with 5% statistical significance. Because this is a randomized pilot study, we are keeping rigorous power at a moderate significance level, and allowing for a very large difference. Analyses will focus on the estimation of means and variances for future trials.

### Confidentiality and data security

Participation in the study is voluntary, and data will be kept confidential. The participants’ full names or personally identifiable information will not be requested on any study materials. All study data will be stored in password-protected computers, and paper records will be stored in locked file cabinets and will continue to be stored securely after the study.

To protect participants’ privacy, a study-specific email account will be generated by the study team without any participant’s personal information. This study-generated email account will be used to register a user account with Fitbit and Abbott/Libre in order to use their smartphone apps. Participants will not need to use their personal email addresses for the Fitbit and CGM apps. All Fitbit data will be collected by the research staff through a third-party company, Fitabase. Fitabase may have access to Fitbit device-collected activity data and the study-generated email addresses.

## Discussion

Physical activity plays an important role in energy balance and obesity, which is an independent risk factor for cancer recurrence and mortality [72]. It has been estimated that cancer survivors who increased their physical activity from pre- to post-diagnosis by any level had a 39% risk reduction in total mortality [3]. This study will use an innovative approach to motivate cancer survivors to adopt and maintain an active lifestyle and will explore a novel mediator (daily glucose pattern) of the association between physical activity and cancer-related biomarkers.

The wearable devices (Fitbit and CGM) may present technical obstacles to some participants. To minimize this potential issue, we will provide extensive technical assistance as needed. We will provide in-depth illustrated instructions with troubleshooting tips and frequently asked questions for participants to take home, and we will be available by email and phone for technical consultation throughout the study period. Because both Fitbit and CGM are consumer-facing devices, they are designed to be easily set up and used with a minimum of support needed. In our previous studies, participants reported few problems, so we anticipate that technical issues will not reduce feasibility. We will closely monitor Fitbit and CGM adherence (through the Fitabase and LibreView platforms, respectively) and send reminders to participants if we detect low adherence to the study protocol. We aim to minimize attrition by scheduling in-person appointments at convenient times for participants (e.g., evenings or weekends, if needed) and providing compensation to participants. These approaches in previous studies have successfully kept attrition rates low. We will monitor study drop-out throughout the study period. If we observe a higher-than-expected drop-out rate, we will examine predictors of study drop-out and explore additional strategies to prevent further drop-out. We will compare participants who drop out of the study with those who complete the study. This information will inform the study design consideration for the future larger trial.

This pilot intervention study will provide biological feedback based on CGM data to demonstrate the acute health benefits of physical activity. This type of biological feedback has a high potential to enhance the effectiveness of traditional performance feedback, which is widely used in physical activity interventions. Further, we will test the remote delivery of CGM-based biofeedback, laying the foundation for future work that could deliver personally relevant feedback in a timely manner. The remote monitoring and delivery of intervention materials are particularly important as CGM and related biosensor technologies become more accessible, and data can be accessed and analyzed in real-time. This remotely-delivered intervention strategy could be an alternative or a complement to existing exercise programs for cancer survivors where mostly involve in-person visits to a gym-like environment [73].

## Supporting information

S1 Checklist (DOC)

S1 Protocol (PDF)
