# Supplementary material for: Using wearable biological sensors to provide personalized feedback to motivate behavioral changes: Study protocol for a randomized controlled physical activity intervention in cancer survivors (Project KNOWN)
Source: PLoS One. 2022 Sep 13;17(9):e0274492. doi: 10.1371/journal.pone.0274492 (PMC9469963; doi:10.1371/journal.pone.0274492)
Supplement: S1 Protocol — (PDF) [file pone.0274492.s002.pdf]

UNIVERSITY OF TEXAS ARLINGTON

INSTITUTIONAL REVIEW BOARD (IRB) FOR THE PROTECTION OF HUMAN SUBJECTS
APPLICATION FOR PRIMARY RESEARCH INVOLVING HUMAN SUBJECTS

UTA Faculty, staff, or students who propose to engage in any research, research development, testing or evaluation with human subjects must have review and approval from the UTA IRB prior to initiation. Some activities involving humans are not considered human subject research requiring IRB review (i.e., class projects, program evaluation, oral histories, quality improvement). Refer to the ***Research Project Chart*** *for more information.*

Utilize the* ***Required IRB Documents Chart *to guide you through the full IRB application process.* ***All study personnel must have completed Human Subjects Protection (HSP) Training prior to study approval. HSP Training expires and must be retaken every 3 years.*****

If you require assistance to complete this form or need additional information, please contact Regulatory Services at 817-272-3723 or regulatoryservices@uta.edu. Regulatory Services also has open office hours every Thursday from 9:00 – 11:00am.

This version of the IRB Application Form should be used for ALL studies that will involve "*primary research*" with human subjects, defined as: the collection of new information or biospecimens from human subjects for research purposes by way of: 1) *interaction* with the individual, which includes any form of communication or interpersonal contact between the investigator(s) and the subject; and/or 2) *intervention* with the individual, which includes both physical procedures by which information or biological samples are gathered (like blood draws) and manipulations of the subject or the subject's environment for the research. Additionally, this form should be completed for studies that involve BOTH primary and secondary research.

IMPORTANT: Studies that will involve only secondary research use of private identifiable information or identifiable biospecimens that have been (or will be) collected or generated for purposes other than the present research study should instead complete the UTA IRB Application for Secondary Research.

SECTION A: GENERAL INFORMATION

1. **Non-UTA Personnel:** *Enter all individuals that are* ***NOT affiliated with UTA*** *who will interact or intervene with human subjects for the research study OR who will access identifiable subject data.* ***UTA-affiliated*** *personnel should be listed on the electronic portion of the protocol (#3) in the electronic submission system.*

   ****Note:*** *In the electronic submission system, upload a completed Non-UTA Collaborator Form and Human Subject Protection training for each listed Non-UTA individual.*

**Name:**	**Organization:**
Sadia Ali	UT Southwestern

2. **Expected Start Date and Completion Date:** April 15, 2022 – April 30, 2025 **(pending approval)** *(You are not authorized to start any research on human subjects including subject recruitment until the IRB has approved the research protocol.)*

3. **Funding:** *Indicate existing, potential, or pending sources of funding below (you may select more than one).*
   ***Note: If you do (or may) receive funding from NSF, NIH, CMMS, DOD, DOJ, DOE, DOEd, DOT, or any other federal agency, you MUST disclose this funding source below to ensure that your study is reviewed in accordance with the appropriate federal regulations for that specific federal funding source.**

External:

☐ **Federal (Sponsor:)** ☐ **State (Sponsor:)** ☐ **Industry (Specify Sponsor:)**

Other (Foundation): American Institute for Cancer Research

Grants & Contracts Bluesheet Number from Mentis: 2021-683

Other:

☐ **UTA Department Account** ☐ **Personal Funds** ☐ **Other:** ☐ **None (*No funding*)**

SECTION B: RESEARCH CLASSIFICATION, RATIONALE, PROCEDURES, SITES, QUALIFICATIONS, OVERSIGHT

4. **Research Classification:** *Indicate if this study is categorized as* ***Minimal Risk (MR)*** *or* ***Greater than Minimal Risk (GMR).*** *"Minimal Risk (MR)" means that the probability and magnitude of harm or discomfort anticipated in the research are not greater in and of themselves than those ordinarily encountered in the subjects' daily life or during the performance of routine physical or psychological examinations or tests. "Greater than Minimal Risk (GMR)" refers to research activities that do not meet the definition of "Minimal Risk." Throughout this application form, there are additional questions or information requested for studies categorized as GMR; these instructions will be presented in purple.*

☒ **Minimal Risk (MR)** ☐ **Greater than Minimal Risk (GMR)**

***Note:** Studies that are federally funded and/or FDA regulated will be further classified into exempt, expedited, or full board in accordance with the Common Rule 45 CFR 46 and/or 21 CFR parts 50 and 56. See Flowchart.*

5. **Rationale:** *List the primary research questions, hypotheses, and / or objectives guiding this study.*

Considerable epidemiologic research suggests that physical activity can reduce cancer-related and overall mortality in survivors of several cancers.[1,2] Randomized clinical trials have also provided strong evidence that physical activity after cancer diagnosis offers significant improvement in survivors' physical outcomes (e.g., increased fitness and physical functioning, decreased fatigue) and mental health (e.g., increased self-esteem, decreased depression and anxiety). This scientific evidence of the numerous benefits of physical activity has resulted in an updated exercise guideline for cancer survivors in 2019 , which calls every survivor should engage in adequate physical activity.[3]

Possible biological mechanisms through which physical activity could affect cancer progression include decreased levels of circulating sex hormones (e.g., estrogens, androgens), reduced systemic inflammation, and positive changes in metabolic markers (e.g., decreased insulin and glucose levels).[4] Chronic and acute hyperglycemia could have downstream effects on cell proliferation and tumorigenesis, possibly via modulation of the insulin-like growth factor (IGF) axis.[5] Previous physical activity interventions showed beneficial changes in biomarkers implicated in these pathways, including insulin, leptin, IGFs, and C-reactive protein (CRP) in cancer survivors.[6–8] In overweight and obese adults, a significant decrease in inflammatory biomarkers such as interleukin-6 (IL-6) and tumor necrosis factor alpha (TNF-α) was found after a 12-week aerobic exercise intervention[9] and a 16-week low-intensity, internet-delivered physical activity intervention.[10] Nevertheless, despite the evidence of the beneficial effects of physical activity, approximately 84% of cancer survivors are not sufficiently active in their daily lives.[11,12] Therefore, to improve post-treatment cancer outcomes and quality of life for cancer survivors, it is vital to promote an active lifestyle for this population through effective behavioral interventions.

One of the key behavioral change strategies in physical activity intervention is the delivery of performance feedback.[13] Several behavioral change theories advocate the use of performance

feedback, positing that feedback on current performance relative to behavioral goals motivates behavioral change.[14] However, the effects of performance feedback on behavioral changes are inconsistent.[15,16] One reason for this inconsistency could be that performance-based feedback alone may not be sufficiently motivating. In particular, physical activity is often characterized by the expense of immediate effort without a tangible short-term benefit.[17] Thus, to enhance the effectiveness of providing feedback to motivate physical activity, there is a critical need to develop methods that could help individuals grasp the health benefits of physical activity in a more immediate and concrete way.

Providing feedback on individuals' biological indices has been used to increase motivation and promote behavior change in the past.[18] In fact, biofeedback is one of the behavioral change techniques under the category of feedback and monitoring.[19] However, biofeedback has not been fully utilized in physical activity interventions. **Given the rapid advancement in wearable sensor technologies that has made continuous monitoring of personal biological data more accessible, we see a great opportunity to further develop and apply personalized biofeedback in physical activity interventions for cancer survivo**rs. Specifically, we could incorporate performance-based feedback typically provided in a physical activity intervention into a biological outcome that is favorably and acutely affected by physical activity to increase motivation for behavioral changes. For such feedback to be personally relevant for cancer survivors, the candidate biological outcome must have long-term clinical implications for cancer survivorship and disease outcome. Therefore, we propose to use glucose data as the basis for providing such biofeedback in cancer survivors to motivate physical activity for two main reasons: First, acute bouts of physical activity can improve insulin sensitivity and increase glucose uptake by skeletal muscles[4] (i.e., the immediacy of the physical activity effects). Second, cancer survivors are at a high risk of developing T2D compared to those without cancer diagnosis[20–22], and elevated blood glucose in nondiabetic cancer survivors has been associated with poor prognosis[23,24] (i.e., the relevance of glucose data to cancer survivors). In cancer survivors, T2D is one of the most common comorbid conditions. T2D or impaired glucose tolerance has been found to be associated with increased cancer mortality, especially in the physically inactive population.[4]

In recent years, an increasing number of studies have used continuous glucose monitors (**CGMs**), which measure glucose concentrations in the interstitial fluid in real-time through a tiny sensor inserted under the skin, to obtain more frequent readings (e.g., every 5-15 minutes) of glucose data to better illustrate this acute impact of physical activity on insulin sensitivity and glucose metabolism in controlled laboratory settings.[25–28] Few studies, however, have used CGMs outside of laboratory settings to provide feedback on the immediate benefits of physical activity on daily glucose patterns. Preliminary data from our research group show that physical activity interventions featuring the use of CGMs are highly feasible and acceptable in sedentary overweight and obese adults.[29] Our qualitative studies with breast and colorectal cancer survivors also indicate high acceptability of CGM-based biofeedback to promote physical activity.[30] Taking together, **utilizing data from CGM to provide personalized biofeedback is highly promising in relaying the immediate benefits of physical activity and the long-term health outcomes for insufficiently active cancer survivors who are at high risk for T2D.** This line of research (i.e., leveraging biosensor data to deliver personalized and timely feedback messages to motivate physical activity) has great potentials in scaling up in the future as there are already several startup companies offering CGM directly to consumers for personalized nutrition purpose (despite lack of incorporating evidence-based behavioral change strategies).[31]

Furthermore, previous studies have found that physical activity and chronic hyperglycemia (i.e., elevated hemoglobin A1c) are correlated with certain cancer-related biomarkers (e.g., insulin-related pathways and inflammation).[5] However, little is known about whether daily glucose patterns (e.g., 24-h average, glucose variability, acute hyperglycemia) are associated with these cancer-related biomarkers in cancer survivors. Thus, identifying daily glucose patterns that might serve as mediators

of the association between physical activity and changes in cancer-related biomarkers will lead to practical implications for cancer survivorship care and program design.

The objectives of this project are:

Objective 1: Evaluate the feasibility of a wearable sensor-based, biofeedback-enhanced 12-week physical activity intervention in insufficiently active (< 90 minutes of aerobic exercise per week) post-treatment cancer survivors who are at high risk for T2D. The intervention will be considered feasible if ≥ 80% of participants in the intervention group are adherent to the self-monitoring protocol and ≥ 80% of participants complete the post-intervention assessment.

Objective 2: Assess the preliminary efficacy of the intervention on daily physical activity levels by comparing the two study group participants before and after the intervention. We hypothesize that participants in the intervention group will have a greater increase in daily physical activity levels over time compared to participants in the control group.

Objective 3: Explore the association between daily glucose patterns (e.g., 24-h average, daily variability, time in hyperglycemic zone) and circulating markers of insulin-related pathways, inflammation, and metabolism such as glycosylated hemoglobin (HbA1c), insulin, IGF-1, IGF-2, IGFBP-1, IGFBP-2, CRP, leptin, IL-6, TNF-α.

6. **Procedures:** *Describe the procedures step-by-step, including details on all methods that will be used to collect human subject data from the beginning to the end of the study. Describe what data will be collected (and if it will be individually identifiable); when and where the data will be collected; and how it will be collected (instruments or other measures). Use clear, concise layman's language that can be easily understood by persons outside your field and provide definitions for any technical terms. Add pictures if needed. If applicable, description and source of secondary research use of information and/or Specimens.* ****Note: Refer to the Types of Research guidance page for a list of specific information required for different types of research.*** *For GMR research, it is also helpful to provide references or pilot data to support the proposed procedures.*

Recruitment Procedures

We will recruit local (i.e., those who live in the Dallas-Fort Worth metroplex area) post-treatment cancer survivors using various recruitment strategies such as, collaboration with physicians from UT Southwestern, research study recruitment website/database (e.g., ResearchMatch), social media, tabling at survivorship events, and posting notifications in newsletters and Facebook pages of survivorship organizations. Interested individuals will complete a screener (**Appendix A**) to determine eligibility. The eligibility screener can be completed via an online survey using the institution approved QuestionPro online survey software. A study personnel can also go over the eligibility screener questions with the prospective participant over the phone or in person. Once eligibility is confirmed, we will schedule the prospective participant for their first in-person visit at UTA.

Data Collection Procedures

Data collection procedures will take place in-person at UTA campus. Our study is considered as a Phase 2 research study. Whenever UTA decides to adopt COVID-19 restrictions, we will adhere to the COVID-19 guidelines as outlined by UTA, such as the use of face coverings (see **Appendix B** – Request to Conduct HSR During COVID-19 for details). When following procedures related to the COVID-19 restrictions, all study participants who are scheduled to visit UTA campus will need to complete the coronavirus self assessment via QuestionPro, as published by the UTA Health Services (**Appendix C**). Those who answer "yes" to any of the self assessment questions will be rescheduled. We will only follow this additional screening procedure if and when UTA decides to adopt COVID-19 restrictions.

On Campus Visit 1 (Baseline Assessment)

Eligible participants will be scheduled for an in-person visit at Dr. Liao's research lab, the Physical Activity and Wearable Sensors (PAWS) Research Laboratory, located in the SEIR building at UTA (room number 102). At this in-person visit, participants will be asked to provide consent for enrollment in the study. Participants will be required to come to this visit fasted (i.e., no consumption of any foods or beverage except water in the past 8 hours) for their blood draw. This baseline visit consists of four major components and one optional component. This visit will take approximately 1-1.5 hours to complete.

1) Participants will complete a set of questionnaires via QuestionPro assessing demographics (e.g., age, gender, race and ethnicity, marital status, education level, household income, employment status), quality of life,[32] health literacy,[33,34] health information technology use, and physical activity-related psychosocial variables such as self-determination motivation,[35] stages of change,[36] and outcome expectancy.[37,38] (**Appendix D**) Participants will complete an online dietary questionnaire via VioScreen, a comprehensive dietary questionnaire, management and analysis system used by healthcare professionals.[39]
2) Fasting blood samples will be collected by a phlebotomy trained research personnel to assay biomarkers (e.g., metabolic markers, lipid panel, inflammatory markers, and insulin-related pathway markers). The amount of each blood draw will be approximately 3-4 tablespoons (e.g., 40-60 milliliter). Collected de-identified blood samples will be stored in a -80 degree freezer on the 1st floor clinical space of SEIR building until analysis. All refrigerators and freezers for blood sample storage are located in a card-accessed space, which have locks and temperature monitor system. Basic commercially available biomarker analysis (e.g., metabolic markers such as glucose, HbA1c and insulin, lipid panel, and c-reactive protein) will be performed by LabCorp Inc. (the same facility that processes blood samples from Student Health Center at UTA and other SEIR research labs). Each blood sample container is labeled with two identifiers, the subject initials+study number, and the subject's date of birth, as required by LabCorp (e.g., Subject: ABC 01234, DOB 10/14/65) and placed in a locked drop-box. LabCorp will be notified, and a courier will retrieve the sample from the locked drop-box on the 1st floor lobby outside of the clinical space of the SEIR building the same day. A portion of serum with de-identified study number only label will be analyzed for other metabolic, inflammatory markers and insulin-related pathway markers (e.g., leptin, IL-6, TNF-α, IGF-1, IGF-2, IGFBP-1, IGFBP-2) will be analyzed in Pan's lab on 2nd floor of SEIR building. Transportation of the blood samples will follow the procedures listed in the UTA Biosafety Manual (https://www.uta.edu/campus-ops/ehs/biological/docs/biosafety-manual.pdf). Briefly, blood samples will be packaged in a sealed, leak-proof, primary container (e.g., plastic screw cap tube). These primary sample containers will then be positioned securely inside a closable secondary leak proof container (e.g., Ziploc bag) for transport. When transporting the samples from 1st floor to 2nd floor of SEIR building, a container made of sufficient strength to protect the transported specimen (e.g., a cooler or a shipping box) will be used with the PI's name and phone number, and labeled with the biohazard symbol. All stored data are de-identified. No genetic analysis will be performed. Prior to the blood draw, participants will be asked a few questions to verify their fasting status, medications that they have taken in the past 24 hrs, and other behavioral factors that might influence the results of the biomarker assay analysis (**Appendix E**). Participants will also have their height and weight measured by study staff.
3) Participants will be given a triaxial accelerometer (ActiGraph GT3X) and will be instructed to wear it on their non-dominant wrist at all times for the next 2 weeks (see **Figure 1**). The ActiGraph GT3X model is a widely used research-grade accelerometer to measure daily activity levels. The ActiGraph device will automatically record participants' movement

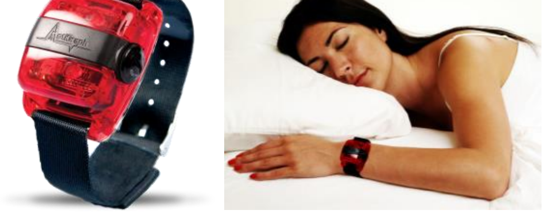

Figure 1. The wrist-worn ActiGraph device (left) measures at 4.6 cm x 3.3 cm x 1.5 cm. A model wears the device while sleeping (right).

data. It is a blinded device so that participants will not be able to view any of their activity data during this baseline assessment period. A fully charged ActiGraph device can continuously record data for up to 25 days. Thus, no interaction is needed from the participants when they wear this device during the 2-week (14 days) baseline monitoring period. The ActiGraph acceleromter is a FDA510(k) cleared Class II medical device, and we will use this device in the context for which it is approved for research purposes (see **Appendix F** for user manual).

4) Participants will be provided with a Freestyle Libre Pro CGM sensor (Abbott Laboratories, California) inserted on the back of their upper arm. This CGM system is FDA-approved and comprised of a sensor and a reader (**Figure 2**). The CGM sensor is designed to be self-inserted into the back of the upper arm. Instructional videos, available from the Freestyle Libre website, will be used to guide the sensor insertion. In Dr. Liao's previous pilot studies, cancer survivors were able to follow the instructions and self-insert the sensor without any major problems. Nevertheless, if preferred by the participant, the sensor can be inserted by our study staff. Once inserted, our study staff will activate the sensor via the provided CGM reader. Once activated, the sensor will start recording interstitial glucose data automatically every 15 minutes continuously for 14 days without the need for any additional user interactions (e.g., finger-stick calibration). The CGM sensor is waterproof. Therefore, once inserted, there will be no need for participants to remove the sensor during the 2-week (14 days) baseline monitoring period. We will apply extra adhesives over the sensor to help secure its position and ensure the continuous collection of glucose data. To blind the glucose data from participants, the CGM reader will not be provided to them. All glucose data are stored in the sensor and will be downloaded by study staff when the assessment period is completed. The Freestyle Libre Pro CGM system is a FDA510(k) cleared Class II medical device (see **Appendix G** for user manual), and we will NOT use this device for clinical purpose (i.e., diabetes management). People with diabetes are not eligible for this study. We do not intent to diagnose any medical condition based on the collected data.

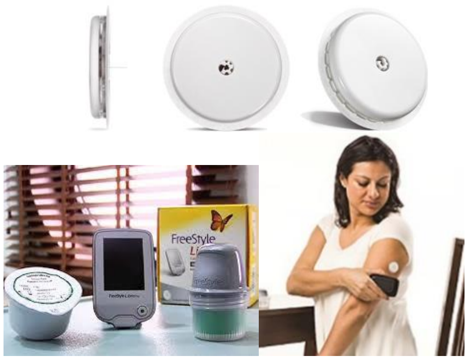

Figure 2. Freestyle Libre sensor (top) and applicator and reader (bottom left). A woman uses the reader to scan the sensor (bottom right).

Optional Procedure: DEXA Scan. Participants can choose if they would want to take part in the DEXA (Dual-Energy X-ray Absorptiometry) scan. We use the DXEA scan to measure lean body mass to determine each individual's body composition, specifically, percentage body fat, visceral fat, and percentage lean mass. The DEXA scan exposes the subject to a minimal amount of radiation which will involve minimal risk to the subject. Anyone that is pregnant or think they may be pregnant are not eligible for this study. A pregnancy test (using an over-the-counter pregnancy test kit) will be performed for women of childbearing age. The DEXA scan will take approximately 15-20 minutes. Participants will be asked to dress in comfortable clothing and to lie down on the table next to the DEXA scanner on their back. The DEXA scanner will begin to slowly move from the top of their head to their feet emitting a very low dosage of x-ray beams. This is how the DEXA will detect how much of the person's body is fat and how much is fat free mass. If a participant chooses to take part in this optional procedure, they will receive a printout of their results at the end of the study period. Since this is an optional procedure, the participant can choose to take the scan during this visit, or during their on campus visit 2 in two weeks.

Baseline Self-monitoring Period (2-week)

Participants are expected to carry out their normal daily activities during this 2-week baseline self-monitoring period. They will keep wearing the ActiGraph device and the CGM sensor at all times during this period. No additional actions are required from them.

On Campus Visit 2 (Intervention Visit)

Participants will be scheduled for their second on campus visit after the 2-week self-monitoring period. Participants will be randomized to either the intervention group or the control group. We will use the minimization method, which randomizes participants based on the assignment that would provide the best overall balance with respect to selected covariates (e.g., cancer type, gender, age, race/ethnicity, and weight category).[40,41] Before a participant is assigned to a group, the number of participants in each group with similar covariate characteristics is totaled. These totals are based on marginal sums of the covariates so that each covariate is considered separately. Participants' assignments are determined based on which group assignment provides the best overall balance with respect to covariates.

First, all participants will return their baseline assessment equipment (i.e., ActiGraph and CGM sensor). Second, all participants will (a) receive an educational handout (**Appendix H**) that discusses prevalent comorbidities in cancer survivors highlighting the T2D risk, the short-term and long-term benefits of physical activity on cancer survivorship, and tips about becoming more active in their daily life; (b) review a published interactive simulator[42] that displays examples of graphs that demonstrate activity-related glucose reductions; (c) go through a worksheet to determine their targeted heart rate zone (**Appendix I**), and (d) set a personalized exercise plan that they feel comfortable to achieve the goal of accumulating 150 minutes of moderate-intensity physical activity each week (**Appendix J**). Third, all participants will be given a Fitbit Inspire 2 fitness tracker to wear during the 12-week intervention period. Participants will be instructed to wear this Fitbit wristband at all times. The Fitbit Inspire 2 is swim-proof and water-resistant to 50 meters. A fully charged Inspire 2 can continuously track activities and heart rate for up to 10 days. We will encourage participants to charge the Fitbit device, as needed, when they take a shower. A Fitbit mobile application (app), available in both Android OS and iOS, will be downloaded to participants' phones. Study staff will teach participants how to keep the Fitbit device synced with the Fitbit app. Upon syncing, study staff will be able to monitor participants' Fitbit information (including activity data, battery status, and syncing events) in real-time through Fitabase (Small Steps Labs LLC, California), a web-based platform that processes Fitbit data and generates activity reports and graphs. Reminders will be sent out if noncompliance (e.g., device non-wear, low battery, outdated syncing) is detected.

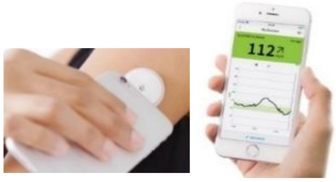

Figure 3. A woman uses a smartphone to scan the Freestyle Libre 2 sensor (left). The LibreLink app displays real-time glucose reading and a trend graph (right).

Participants assigned to the intervention group will be additionally provided with a Freestyle Libre 2 CGM (see **Appendix K** for user manual). They will need to wear this CGM during the first four weeks of the intervention period (details in the section below). This CGM system is similar to the Freestyle Libre Pro used in the baseline assessment period, except that participants will be able to use their smartphone to scan the sensor and obtain their glucose information in real-time through the LibreLink mobile app (see **Figure 3**). Participants can indicate if they want to self-insert the CGM sensor or prefer a study staff to insert the sensor for them. Study staff will help participants download the LibreLink app, available in both Android OS and iOS, and teach them how to use their phone to obtain their glucose information. Upon scanning the smartphone with the sensor, the LibreLink app will display the current glucose reading; a trend arrow indicates the direction the glucose is moving, and a graph shows an 8-hour history of the glucose value. A Freestyle Libre sensor will continuously record interstitial glucose data every 15 minutes for 14 days. Participants will need to replace the CGM sensor once for a total of 4-week monitoring. Participants can have the option of replacing the sensor on their own, or if preferred, they can come back to UTA campus for a quick visit and a study staff will replace the sensor for them.

This intervention visit will take about 30-60 minutes to complete.

Intervention Self-monitoring Period (12 weeks)

All participants in the control and intervention groups will wear the Fitbit Inspire 2 wristband during this 12-week intervention period. Participants can track their daily steps, exercise minutes and intensity, as well as daily activity trends and progress towards goals through the Fitbit app. Participants will be encouraged to engage in at least 150 minutes of moderate-intensity aerobic physical activity each week. All participants will receive educational text messages 2-3 times per week that reflect the topics in the educational handout (e.g., benefits of physical activity for cancer survivorship, tips for exercising, reminders about goals).

Participants in the intervention group will additionally wear a Freestyle Libre 2 CGM during the first four weeks of the intervention period. Participants will be instructed to scan the CGM sensor with their smartphone at least 4 times each day (i.e., upon awakening, afternoon, evening, and before sleep). Each time participants scan the sensor using their smartphone, their glucose data will be synced to a server wirelessly through the LibreLink app. Study staff will be able to access participants' CGM data and daily glucose pattern summary statistics remotely via LibreView, a web-based platform developed by the manufacturer to view participants' CGM data and generate reports. Study staff will reminder participants about sensor scanning if missing scans are detected. During this 4-week CGM monitoring period, participants will receive personalized biofeedback messages 1-2 times per week based on their Fitbit and CGM data. These biofeedback messages incorporate participants' Fitbit and CGM real-time data with topics such as goal-setting, self-monitoring, and outcome expectations (see **Table 1** for sample messages). These biofeedback messages have also been pilot-tested in the target population by Dr. Liao. Data to tailor these messages will be available from the LibreView platform and the Fitabase platform. During the weeks that participants are not wearing the CGM sensor (i.e., weeks 5-12), they will receive messages reminding them of the acute impact of physical activity on their glucose patterns. All messages to be delivered in this study will be managed and sent under a licensing agreement with Mosio (Mosio, Washington), a two-way text messaging company that specializes in HIPAA compliant mobile solutions for clinical research. The study team has been utilizing this mobile messaging platform for previous studies that generate feedback messages using Fitbit and CGM data. For messages that include options for interactive experience sharing (i.e., by replying to the text message), study staff will be able to view and download participant responses through the Mosio platform.

Towards the end of week 4, we will mail all participants the ActiGraph device for them to wear during week 5 (i.e., mid-intervention assessment). Participants will also complete the VioScreen during week 5 for dietary assessment.

Table 1. Sample personalized biofeedback messages for the intervention group

Topic Category	Sample Text Message
**Goal setting**	▪ Your average glucose level from yesterday was 112 mg/dL. Prolonged time in high glucose level (greater than 140 mg/dL for example) could increase your risk of developing type 2 diabetes. Remember that exercise such as brisk walking could have an immediate impact on your glucose level. If you observe a spike in your glucose, try to add some more activity time. Let's see if your average glucose level will be less than 100 mg/dL next week!
**Self-monitoring**	▪ In the past 5 days, you had 3 occasions that your glucose went above 140 mg/dL. Have you observed any behaviors that might make your glucose level spike? Please share with us.
**Outcome expectations**	▪ In the past week, you had 5 occasions that your glucose went above 140 mg/dL and spent a total of 65 minutes above 140 mg/dL. It is normal that your glucose level fluctuates during the day, especially after meals. Keep in mind that whenever you move, your muscles burn blood glucose for fuel. The more you move the more glucose you burn!

On Campus Visit 3 (Post-intervention Assessment)

Participants will be scheduled for their third on campus visit after the 12-week intervention period. During this visit, participants will answer a set of post-intervention survey (**Appendix L**), complete another VioScreen for dietary assessment, have their height and weight measured, have their fasting blood drawn (same procedures as in visit 1), and complete an exit interview (**Appendix M**). The exit interview will be recorded for further qualitative analysis. All participants will be provided with the ActiGraph GT3X and the Freestyle Libre Pro to wear for the next 2 weeks (same procedures as for the baseline self-monitoring period). This visit will take about 60 minutes to complete.

On Campus Visit 4 (Optional)

At the end of the 2-week post-intervention self-monitoring period, participants can have the option to visit the UTA campus one last time to return the study devices (i.e., the ActiGraph GT3X and the CGM sensor), or they can have the option to mail back the study devices. Participants can keep the Fitbit wristband as part of their compensation for participating in this study. They will also receive up to $50 gift card for completing this study. This visit will take about 30 minutes to complete.

7. **Duration:** *Indicate how many participation sessions, interactions, or follow ups are expected for each subject participant, including the amount of time required for each visit and how long their total participation is expected to take (weeks, months, years, etc.) over the entire duration of the study.*

   There are four on campus visits (visit 4 optional). The first visit will take about 1-1.5 hours to complete. The second visit will take about 30-60 minutes to complete, the third visit will take about 60 minutes to complete, and the last visit will take about 30 minutes to complete.

   There are two self-monitoring assessment period, each will take 2 weeks.

   The intervention self-monitoring period takes a total of 12 weeks.

   The whole study period is 16 weeks.

8. **Alternatives to Participation:** *Describe subjects' available options if they choose not to participate in the research study and clarify whether individuals that decline participation will still be subjected to the intervention (even if their data will not be utilized for research purposes). If research involves students, describe their alternatives to obtain course / extra credit if applicable. If research involves a health intervention, clarify whether individuals that decline will continue to receive standard care.*

   There are no alternatives to participation. Participants can stop taking part of the study at any point during the study period. They will be asked to return the Fitbit device (and any other study devices they might have at the time) if they decide to not participate in the study anymore. Participants can receive partial compensation based on their survey completion rate that is explained in the compensation payout document (**Appendix N**).

9. **Location(s) and Site(s):** *Specify all locations where research procedures are expected to take place and which study procedures will take place at each site. Studies that take place online should specify the websites where data will be collected. Describe if any of the research will take place internationally. For multi-site research studies, review the web page for [Collaborative Research](). If any part of this study will be conducted in an institution or location administratively separate from UTA, indicate the institution(s) and upload a site permission letter.*

   All in-person study-related procedures will take place at the UTA campus. No procedures nor participant interactions will take place at another institute or location.

10. **Personnel Qualifications:** *List each member of the research team/personnel and describe 1) their role in the study, and 2) their relevant qualifications, special training, and experience as it pertains to the specific procedures or population of the study. If personnel will receive special training for conducting this study, please describe. If one or*

more personnel do not have any relevant qualifications or experience, please state that; the IRB will consider the risk level of the study and evaluate if additional oversight or input is necessary.
Dr. Liao, the PI, is an experienced behavioral scientist with an emphasis in the use of mobile technologies and wearable sensors. Dr. Liao's study populations include overweight and obese sedentary adults as well as cancer survivors. Dr. Liao has completed Human Subjects Research Training. She will be responsible for all aspects of this study.

Dr. Brannon, the study collaborator, is trained in health communication from CoLA. Dr. Brannon has completed Human Subjects Research Training. Dr. Brannon will assist with the messaging component of this study and assist with qualitative data analysis, interpretation, and reporting.

Dr. Liu, the study collaborator, is a clinical nurse and an assistant professor of nursing at CONHI. She has completed all related training and will assist with participant recruitment and data collection.

Ms. Arena-Marshall, the study collaborator, is a clinical nurse and a clinical assistant professor of nursing at CONHI. She has completed all related training and will assist with participant recruitment and data collection.

Dr. Beg, the study collaborator, is a medical oncologist at UT Southwestern. Dr. Beg has completed Human Subjects Research Training. Dr. Beg will assist with participant recruitment.

Dr. Ali, the study collaborator, is an endocrinologist at UT Southwestern. Dr. Ali has completed Human Subjects Research Training. Dr. Ali will assist with participant recruitment and provide clinical guidance regarding diabetes risks and interpretation of CGM data.

All undergraduate and graduate student research interns and research assistants that are involved in this study have completed Human Subjects Research Training. Students will assist with participant recruitment, data collection, and participant monitoring. These students are:

Mahima Pandya, graduate research assistant – she will help with participant recruitment/screening, data collection, intervention delivery, participant monitoring, and data management/analysis.

Hannah Affleck, graduate research assistant – she will help with participant recruitment/screening, data collection, intervention delivery, participant monitoring, and data management/analysis.

11. **Study Oversight:** *The Principal Investigator has ultimate responsibility for the conduct of this research, protection of subjects, and supervision of all protocol personnel. Describe your plan for oversight and communication to ensure that the entire research team: conducts the research ethically and in accordance with the approved protocol, creates/maintains appropriate study documentation and research records, and protects confidentiality of data.*
Dr. Liao is responsible for overseeing the conduct of this research study and ensuring the protection the research subjects. She will also be responsible for ensuring that research documents (consent) are appropriately documented. Dr. Liao will supervise all protocol personnel.

SECTION C: POPULATION & ENROLLMENT

12. **Population(s):** *Describe the target population(s) of the study, for example: UTA students, competent or healthy adults, children, prisoners, non-English speaking, pregnant women, individuals with impaired decision-making capacity, other vulnerable populations.*
The target population for this study is cancer survivors who are over 18 years of age.

****Note: Additional forms may be required for your population. Obtain these from the Forms & Templates Page.***

For Individuals with Impaired Decision-Making Capacity: Upload Form 2A.
For Pregnant Women, Fetuses, Women Undergoing In-Vitro Fertilization, or newborns: Upload Form 2B.
For Prisoners (Individuals involuntarily detained): Upload Form 2C.
For Children (Under 18 or the local legal adult age): Upload Form 2D.

13. Inclusion Criteria: *List all criteria for including subjects, and explain the methods you will use to determine whether a subject is eligible based on your criteria (i.e. pre-screen, medical chart review). If your study is/will be funded, ensure that the inclusion criteria listed here match the details in your proposal.*
To be eligible for the study, individuals must be 18 years or older, have had a diagnosis of cancer; have completed curative-intended treatment for at least 3 months (except hormone therapy or long-term maintenance chemotherapy); be at high-risk for type 2 diabetes based on the American Diabetes Association Type 2 Diabetes Risk Test[43,44] (a score of 5 or higher based on the seven screening questions), be insufficiently active (engaging in < 90 minutes of moderate-intensity aerobic physical activity per week in the past month); capable of participating in moderate-vigorous intensity unsupervised exercise (either no positive responses on the Physical Activity Readiness Questionnaire,[45] or clearance from [see **Appendix O**] a health care provider certifying that the patient is healthy enough to engage in unsupervised exercise); have no current diagnosis or history of type 1 or 2 diabetes; ability to speak, read, and write in English, and have a smartphone with daily internet access.

14. Exclusion Criteria: *Explain any specific factors or contraindications that would make a subject ineligible to participate in this study, even if they would otherwise meet the inclusion criteria listed above. If your study is/will be funded, ensure that the exclusion criteria listed here match the details in your proposal.*

Individuals will be excluded if they are taking oral antidiabetic agents (OADs), current treatment with any insulin regimen other than basal insulin, e.g. prandial or pre-mixed insulin (short term treatment due to intercurrent illness including gestational is allowed at the discretion for the investigator), pregnancy, on dialysis, self-reported health issues that limit physical activity, work overnight shifts, unwilling to use CGM, current participation in other exercise or weight loss-related program or intervention, currently on a low-carb diet; current use of other implanted medical devices such as pacemakers; do not have a smartphone that is compatible with the Fitbit and the LibreLink apps.

15. Number of Subjects: *Provide the number of subjects (or subject records/data sets) you intend to enroll over the course of the study. This information will be utilized by the IRB to understand the scope and logistics of the study; you may provide a projected range. For secondary research, please describe the number of records to be accessed.*
The target sample size for this study is 50 participants.

***Note:** For MR research, after the protocol is approved, enrollment can exceed the number provided here without submitting a modification to the protocol.*

For GMR research, the proposed number of subjects must be supported by statistical justification and/or references; please provide that information here. Enrollment for GMR research is capped (IRB will approve a specific range or maximum number of participants and enrollment must not exceed that approved number unless the IRB approves a modification request).

16. Recruitment Strategies: *Describe how you will identify and contact potential participants, and how you will obtain their contact information. Upload permission letters/emails as needed from individuals or organizations providing access to private contact information. Upload a copy of all planned recruitment materials (i.e. letters/emails; website/social media posts; printed flyers; telephone scripts; subject pool posts (SONA, Mechanical Turk, Research Match); scripts for recruitment in-person).*

We will recruit local (i.e., those who live in the Dallas-Fort Worth metroplex area) post-treatment cancer survivors using several recruitment strategies. First, our study collaborator, Drs. M. Shaalan Beg and Sadia

Ali will directly refer patients from their clinic and promoting this study among UTSW Medical Center clinicians. Second, we will utilize the Commission on Cancer (CoC) accredited Tumor Registry for the UTSW Simmons Comprehensive Cancer Center and the Dallas County Safety Net Hospital, Parkland Health and Hospital Services to identify potentially eligible patients (e.g., based on diagnosis date). We will also use the electronic health record tools "SlicerDicer" to curate patient information. To further expand our patient pool to include a diverse patient population, additional patients could be recruited from local communities through social media, research study recruitment website (e.g., Research Match), tabling at survivorship events, and posting notifications in newsletters and Facebook pages of survivorship organizations. The study team has successfully recruited a diverse cancer survivor population for previous studies using these established recruitment methods. See **Appendix P** for recruitment materials. Reasons for not interested in the study will be documented (**Appendix Q**).

SECTION D: COMPENSATION AND COSTS

***Note:** You are responsible for maintaining accurate and confidential records regarding payment of your subjects. Per Accounting Services procedures, compensation must be documented for tax purposes using a W-9 form unless an exception is granted by the Accounting department. Obtaining an exception should be considered for cases of sensitive research or when disclosure of a subject's identity would expose them to high risk. Exception requests are submitted through the Business Affairs Exceptions Tracker (BAET) in SharePoint. Contact Business Technology Services at 817-272-2155 or submit a ServiceNow ticket at https://uta.service-now.com/selfservice/ for assistance.*

17. Compensation: *Describe any compensation to subjects for participation, including monetary payments, gift cards, course/extra credit, raffle prizes, goods or services, donations to charity, etc. Describe how and when you will provide the payment to the subjects, and how confidentiality will be maintained (for example, use of coding in payment log books/receipts). If you intend to hold a raffle, explain when you expect that the raffle will be drawn, and how participants will be contacted if they win the drawing. For course / extra credit, alternative non-research assignments must be offered for an equal amount of credit.*

Participants can keep the Fitbit wristband and receive up to $50 in gift card upon the successful completion of this study. Participants' compensation amount will be based on their completion of required study components (**Appendix N**) to encourage compliance. Gift card will either be given to the participant in person or be electronically sent to them at the end of the study period.

18. Costs: *Describe any costs or expenses (monetary or non-monetary) subjects will incur as a result of participation.*

There will be no cost for participate to take part in this study. Participants will need internet access to use the Fitbit app and the LibreLink app using their smartphones. Participants will receive multiple SMS text messages sent to their mobile phones each week. They may also reply to those text messages using their mobile phones.

SECTION E: INFORMED CONSENT

***Note:** The ethical foundation of human subject research is informed consent. It is important to ensure that subjects are provided with sufficient information to understand the requirements of their participation and the use/purpose of their data. You also cannot obtain information about a person through another individual (such as a family member) unless that person has undergone the informed consent process themselves. Use the Office of Human Research Protection (OHRP) informed consent checklist (http://www.hhs.gov/ohrp/policy/consentckls.html) and the IRB's Templates as guidance.*

19. Informed Consent, Broad Consent, & Assent: *Describe the informed consent process, including when, where, and how subjects will be consented. If children or mentally disabled or incapacitated persons will be subjects, explain the assent process. If broad consent (consent to use data for future studies) will be requested, describe the scope and the process for tracking subjects' accept/decline responses. Upload finalized copies of all consent, assent, and / or verbal*

consent script documents in the electronic system. If applicable, please address informed consent for any secondary research. ***There are several consent form templates available for your use on the [Forms & Templates Page](#).***

At the beginning of their first in-person visit, each participant will receive a verbal description of the study protocol, methodology, risks, and compensation. They will have the opportunity to read through the consent form and ask any questions they might have. Upon agreement of enrollment, each participant will provide their written informed consent by signing the Informed Consent Document. A copy of the signed consent form will be provided to each participant.

19a. Requesting a Waiver of Consent or Waiver of Written Documentation: *If you wish to waive some or all of the requirements of informed consent, or the requirement for written/signed informed consent, please describe (if your study is federally funded or FDA-regulated, also upload Form 3 from the Forms Page.*

20. Incomplete Disclosure or Deception: *Describe if your study will withhold information (incomplete disclosure) from subjects or involve deception regarding the purpose of the research or the nature of the intervention, interaction, or procedures. Provide scientific justification for utilizing incomplete disclosure or deception (if your study is federally funded, also upload Form 3).*

N/A

****Note:** "Incomplete disclosure" occurs when an investigator withholds information about the specific purpose, nature, or other aspect of the research. "Deception" occurs when an investigator gives false information to subjects or intentionally misleads them about some key aspect of the research.*

SECTION F: RISKS & BENEFITS

21. Risks to Subjects: *Explain any potential risks to subjects that could result from the research intervention/procedures, including **physical risks** (i.e. fainting, falls, infections, muscle soreness, pain, broken bones, physical fatigue, headache, burns, medication side effects); **psychological risks** (i.e. depression, anger, stress, guilt, embarrassment, damage to self-esteem); **social risks** (i.e. potential damage to financial standing, reputation, or employability); **risks to privacy or confidentiality** (i.e. exposing someone as a research subject, release or breach of sensitive data); and/or **risk of perceived coercion/undue influence** (i.e. if investigator could have influence by nature of their relationship or status, such as a teacher & student, manager & employee, doctor & patient).*

Physical Risks

Potential physical risks to participants are predominantly associated with the CGM. Because we will be excluding individuals with diabetes (Type I or II), we do not anticipate that any of these risks will be associated with the inaccuracy of the blood glucose reading (e.g., inaccurate detection of dangerously low or high blood glucose levels). Rather, potential risks are most likely to be associated with inserting the sensor, including discomfort in the area the sensor is inserted. Per the Abbott Freestyle Libre User's Guide and FDA documents, some individuals may be sensitive to the adhesive that keeps the sensor attached to the skin. There is a reportedly low chance of this happening as clinical studies for the CGM indicated that only slight redness and swelling occurred in a few patients. Pain was mostly reported as none. Lastly, sensor breakage with fragments retained under the skin could be another complication. However, based on post-market experience and clinical studies, this event is rare and its severity does not raise major concerns.

Psychological/Emotional Risks

Psychological/emotional risks are minimal and might include reminding participants of their concerns and challenges dealing with their physical activity and daily symptoms/experience.

Social Risks

There are no anticipated social risks associated with this study.

Risks to Privacy or Confidentiality
There is a risk of breach of confidentiality that could reveal the identity of the research participant. Only the study personnel will have access to the lab email (pawslab@uta.edu), which will be used for communication with prospective study participants. Text messages are being sent through a third-party company, Mosio, that may have access to the content of the messages and participant phone numbers. Participant's full name or other identifiable information will not be disclosed to Mosio.

Risk of Perceived Coercion/Undue Influence
Risk of perceived coercion/undue influence is low as there is no relationship between the principal investigator and research subjects. There is a minimal risk of participants' social desirability bias given the principal investigator's status as "researcher." It is also possible that participants may be suspicious of participating in this study out of concern it might be used by "the government."

22. **Strategies to Minimize Risks:** *Explain the strategies that the research team will use to minimize the potential risks listed above.*
Physical Risks
We will closely follow all procedures outlined in the Freestyle Libre user manuals for sensor insertion. This CGM system is designed to be self-inserted. We will provide guidance to participants (with instructional videos provided by the manufacturer, and if they prefer, we can insert the sensor for them. During the CGM wear-time, participants are encouraged to contact the study staff for any concerns. Participants will also have the option to contact their own healthcare provider and provide him/her with the Abbott Technical Support contact information. Following these strategies, in Dr. Liao's previous studies that utilized CGM systems, no severe concerns or incidents were reported by study participants.

Psychological/Emotional Risks
Although the principal investigator anticipates minimal emotional discomfort/risk, to further minimize potential risk, participants will be able to skip any questions that they may feel uncomfortable to answer.

Risks to privacy or confidentiality
All electronic data downloaded from QuestionPro (a UTA-sponsored account) will be de-identified (i.e., deleting IP address). No identifiable data will be collected by the Actigraph device, the CGM device, or the Fitbit device. We will create a study-specific email account without any participant's personal information for them to register a user account with Libre and Fitbit in order to use their smartphone apps. All study data will be stored in a password-protected OneDrive file folder on a password-protected UTA encrypted computer. To reduce the risk to privacy or confidentiality, the research team will ensure that only the study team members have access to study data and ensure that care is taken to de-identify the dataset.

Risk of perceived coercion/undue influence
To reduce this risk, the Principal Investigator will emphasize in the consent form that there is no conflict of interest and that this research is for academic purposes.

23. **Health & Safety Considerations:** *Specify whether the study involves any hazardous materials, locations, or equipment that is relevant to the health and safety of either the subjects or the protocol personnel (i.e. handling of human blood/body fluid/tissue, chemical or biological hazards, radiation/X-rays, lasers, or carcinogens). List any related authorizations/approvals from the Environmental Health & Safety Office.*
Blood samples will be collected, stored, and disposed of in accordance with EH&S policies as for other UTA approved protocols. Briefly, based on the EH&S policies for disposal (bio-waste-disposal-instructions.pdf (uta.edu)), we will dispose blood samples inside red autoclavable biohazard waste bag, which is inside a rigid red biohazard waste container. Once the rigid plastic bins are full, they will be autoclaved. The autoclaved bag will then be placed in a black bag and disposed in the trash dumpster. Additionally, all

stored blood samples will be kept in biohazard labelled freezers in the clinical space of the SEIR building.

24. **Benefits:** *List potential benefits that may accrue directly to the study subjects as a result of their participation, if any (other than compensation). Also describe the expected or potential benefits of this study to the field or society at large.*
Participants may start adopting a healthier lifestyle (e.g., increasing their physical activity levels) from participating in the study. Future cancer survivors may benefit from what is learned from this study.

SECTION G: PRIVACY & CONFIDENTIALITY

25. **Privacy:** *How will the privacy of subjects be protected during the course of the study (privacy refers to controlling the environment and circumstances of interactions with subjects to prevent situations where they might be embarrassed, exposed, or stigmatized)?*
The privacy of the participant will be ensured as a result of the record keeping process to be employed. All data collected on individual participant will only be viewed by the investigative team involved in the project (i.e., principal investigator, research assistant, research fellows). The individual data for each participant will be analyzed and stored by study ID, and at no time will any individual data point be identified with a particular individual subject. The data will almost exclusively be presented as average group differences. All data and information regarding the study will be kept in the personal, well-secured laboratory of the principal investigator.

26. **Confidentiality & Data Security:** *Explain if the data collected (including biospecimens) will be anonymous, identifiable/coded, or de-identified*. Explain the precautions that will be taken to protect confidentiality of subject data and information, and how these precautions will be communicated to subjects (during informed consent or another process). Security should be considered for each phase of data's life cycle, including: collection, transmission, accessing, collaboration, storage, analysis, reporting, and disposition. Consider the tools and resources that will be utilized for data collection, how access to identifiable data will be limited only to authorized research personnel, and who will be responsible for storage and disposition.* ***Recordkeeping:*** *UTA and the IRB must be able to access research records and consent forms at any time; therefore,* ***all paper documents in their original form must be stored on the UTA campus*** *unless the IRB grants an exception.* ***All electronic data must be maintained on UTA servers utilizing sanctioned storage tools*** *unless the Office of Information Security grants an exception through the Technology Approval Process (TAP).* ***Record Retention Period:*** *All records (paper or electronic) must be maintained and kept secure for at least 3 years after the closure of the protocol or in accordance with funding agency requirements (whichever is longer). Student PIs should address long-term storage arrangements if planning to leave UTA prior to the end of the retention period.*
Visit the UTA IRB's Web Page on Human Subjects Data Security for allowable data storage options and more helpful information about DO's and DON'Ts with human subject data! To create a TAP Request, go to https://uta.service-now.com/selfservice2 , select "Request Something", select "Technology Approval Process" under the section "Desktop or Lab Hardware & Software", then complete questions for the TAP review.
Participation in the study is voluntary and data will be kept confidential. The participants' full names or personally identifiable information will not be requested on any study materials. All study data will be stored in password-protected computers and paper records will be stored in locked file cabinets and will continue to be stored securely after the study.

A study-specific email account will be generated by the study team without any participant's personal information. This study-generated email account will be used to register a user account with Fitbit and Abbott/Libre in order to use their smartphone apps. Participants will not need to use their personal email address for the Fitbit and LibreLink apps. All Fitbit data will be collected by the research staff through a third-party company, Fitabase. Fitabase may have access to Fitbit device collected activity data and the study-generated email addresses.

Each participant will be assigned a study ID that links their QuestionPro, ActiGraph, CGM, Fitbit, and biomarker assay data. Participants' name and contact information will be stored in a separate password-

protected file and will not be revealed in any study materials. All study data will be stored in password-protected computers on the UTA campus and/or a secure UTA server and any paper records will be stored in locked file cabinets on the UTA campus and will continue to be stored securely after the study.

***Note:** "Anonymous" means that the data is unidentifiable (personally identifiable information will not be collected or accessed). "Identifiable" means that data obtained will be recorded in such a manner that subjects' identity can be readily ascertained, either directly or indirectly through identifiers linked to the subjects (research involving a coding mechanism that links to identifiable data is considered identifiable, but it is a helpful measure to protect confidentiality). "De-identified" means that all direct personal identifiers are permanently removed, no code or key exists to link the data to its original source, and the remaining information cannot reasonably be used by anyone to identify the source.*

26a. Legal Limits to Confidentiality: *If any part of this study could result in the potential identification of child abuse, elderly abuse, communicable diseases, or criminal activities that would / could not have been otherwise identified, explain this possibility and estimate the likelihood of disclosure. Describe the plan of action that you will take if this occurs. In rare circumstances when research reveals these issues, confidentiality should be maintained to the extent that the law allows.*
n/a

Data Sharing: *If you intend to share, release, or present any identifiable subject data from this study, explain where, when, and to whom the identifiable information will be shared, presented or released, and how this will be communicated to the subjects beforehand.*
We will not need to share data outside of UTA. All data gathered in this protocol will be stored in a password-protected database and/or a secure UTA server. Access to the database is only available to individuals directly involved in the study. Information gathered for this study will not be reused or disclosed to any other person or entity, or for other research.

SECTION H: CONFLICT OF INTEREST

27. Conflicts of Interest (COI): *Does the Investigator or any protocol personnel have an affiliation, arrangement, or financial interest that could be perceived as a conflict of interest? If yes, please describe.*
All study team members do not have affiliations that could be perceived as a conflict of interest.

***Note**: "Financial Interest" is defined as anything of monetary value (existing or potential), whether or not the value is readily ascertainable. "Conflict of Interest" is defined as a significant financial interest that could directly and significantly affect the design, conduct, or reporting of research.*

***Note:** All Covered Individuals in GMR research are required to have a current COI disclosure on file in Mentis (this must be complete prior to approval of the protocol). Covered Individuals are those with responsibilities for the conduct, design, or reporting of this research study.*

SECTION I: REQUIRED ADDITIONAL ATTACHMENTS

28. Upload finalized versions of the following documents as applicable to your study in the electronic submission system*:*

- Survey instruments / questionnaires (and any versions translated into other languages)
- Demographics surveys
- Interview questions / prompts
- Focus group instructions / questions / prompts
- Observation data collection sheets

- Psychological & educational tests
- Educational materials
- All recruitment materials including flyers, ads, scripts, emails, social media posts, etc.
- Informed Consent Documents / cover letters and translated versions (See Forms Page for Templates)
- Permission letters from non-UTA study sites / collaborating organizations
- Signed Non-UTA Collaborator Forms & HSP Training (Collaborative Research Page).

REFERENCES

1. Gunnell AS, Joyce S, Tomlin S, et al. Physical Activity and Survival among Long-term Cancer Survivor and Non-Cancer Cohorts. *Front Public Health*. 2017;5. doi:10.3389/fpubh.2017.00019
2. Friedenreich CM, Stone CR, Cheung WY, Hayes SC. Physical Activity and Mortality in Cancer Survivors: A Systematic Review and Meta-Analysis. *JNCI Cancer Spectr*. 2020;4(pkz080). doi:10.1093/jncics/pkz080
3. Campbell KL, Winters-Stone KM, Wiskemann J, et al. Exercise guidelines for cancer survivors: consensus statement from international multidisciplinary roundtable. *Med Sci Sports Exerc*. 2019;51(11):2375-2390. doi:10.1249/MSS.0000000000002116
4. McTiernan A. Mechanisms linking physical activity with cancer. *Nat Rev Cancer*. 2008;8(3):205-211.
5. Lodhia KA, Tienchaiananda P, Haluska P. Understanding the key to targeting the IGF axis in cancer: A biomarker assessment. *Front Oncol*. 2015;5:142.
6. Winzer BM, Whiteman DC, Reeves MM, Paratz JD. Physical activity and cancer prevention: a systematic review of clinical trials. *Cancer Causes Control*. 2011;22(6):811-826.
7. Ballard-Barbash R, Friedenreich CM, Courneya KS, Siddiqi SM, McTiernan A, Alfano CM. Physical activity, biomarkers, and disease outcomes in cancer survivors: a systematic review. *J Natl Cancer Inst*. 2012;104(11):815-840.
8. McDermott. Biological markers as an outcome measure of exercise in cancer rehabilitation: A systematic review. https://www.cancerjournal.net/article.asp?issn=0973-1482;year=2018;volume=14;issue=2;spage=267;epage=277;aulast=McDermott. Accessed May 5, 2021.
9. Farinha JB, Steckling FM, Stefanello ST, et al. Response of oxidative stress and inflammatory biomarkers to a 12-week aerobic exercise training in women with metabolic syndrome. *Sports Med-Open*. 2015;1(1):19.
10. Smith DT, Carr LJ, Dorozynski C, Gomashe C. Internet-delivered lifestyle physical activity intervention: limited inflammation and antioxidant capacity efficacy in overweight adults. *J Appl Physiol*. 2009;106(1):49-56.
11. Friedenreich CM, Neilson HK, Farris MS, Courneya KS. Physical activity and cancer outcomes: A precision medicine approach. *Clin Cancer Res*. 2016;22(19):4766-4775.
12. *National Cancer Institute. Cancer Survivors and Physical Activity*. https://progressreport.cancer.gov/after/physical_activity.
13. Olander EK, Fletcher H, Williams S, Atkinson L, Turner A, French DP. What are the most effective techniques in changing obese individuals' physical activity self-efficacy and behaviour: A systematic review and meta-analysis. *Int J Behav Nutr Phys Act*. 2013;10(1):29.
14. Michie S, Abraham C. Interventions to change health behaviours: Evidence-based or evidence-inspired? *Psychol Health*. 2004;19(1):29-49.
15. Kluger AN, DeNisi A. The effects of feedback interventions on performance: A historical review, a meta-analysis, and a preliminary feedback intervention theory. *Psychol Bull*. 1996;119(2):254-284.
16. Kramer JN, Kowatsch T. Using feedback to promote physical activity: The role of the feedback sign. *J Med Internet Res*. 2017;19(6):192.
17. Orji R, Vassileva J, Mandryk R. Towards an effective health interventions design: An extension of the health belief model. *Online J Public Health Inform*. 2012;4(3):4 3 4321.
18. McClure JB. Are biomarkers useful treatment aids for promoting health behavior change?: An empirical review. *Am J Prev Med*. 2002;22(3):200-207.

19. Michie S, Richardson M, Johnston M, et al. The behavior change technique taxonomy (v1) of 93 hierarchically clustered techniques: building an international consensus for the reporting of behavior change interventions. *Ann Behav Med Publ Soc Behav Med*. 2013;46(1):81-95. doi:10.1007/s12160-013-9486-6
20. Hwangbo Y, Kang D, Kang M, et al. Incidence of diabetes after cancer development: A Korean national cohort study. *JAMA Oncol*. 2018;4(8):1099-1105.
21. Singh S, Earle CC, Bae SJ, et al. Incidence of diabetes in colorectal cancer survivors. *J Natl Cancer Inst*. 2016;108(6):402.
22. Lipscombe LL, Chan WW, Yun L, Austin PC, Anderson GM, Rochon PA. Incidence of diabetes among postmenopausal breast cancer survivors. *Diabetologia*. 2013;56(3):476-483.
23. Monzavi-Karbassi B, Gentry R, Kaur V, et al. Pre-diagnosis blood glucose and prognosis in women with breast cancer. *Cancer Metab*. 2016;4(1):7.
24. Kakehi E, Kotani K, Nakamura T, Takeshima T, Kajii E. Non-diabetic glucose levels and cancer mortality: A literature review. *Curr Diabetes Rev*. 2018;14(5):434-445.
25. Van Dijk JW, Manders RJF, Tummers K, et al. Both resistance-and endurance-type exercise reduce the prevalence of hyperglycaemia in individuals with impaired glucose tolerance and in insulin-treated and non-insulin-treated type 2 diabetic patients. *Diabetologia*. 2012;55(5):1273-1282.
26. Nygaard H, Rønnestad BR, Hammarström D, Holmboe-Ottesen G, Høstmark AT. Effects of exercise in the fasted and postprandial state on interstitial glucose in hyperglycemic individuals. *J Sports Sci Med*. 2017;16:254-263.
27. Crespo NC, Mullane SL, Zeigler ZS, Buman MP, Gaesser GA. Effects of standing and light-intensity walking and cycling on 24-h glucose. *Med Sci Sports Exerc*. 2016;48(12):2503-2511.
28. Peddie MC, Bone JL, Rehrer NJ, Skeaff CM, Gray AR, Perry TL. Breaking prolonged sitting reduces postprandial glycemia in healthy, normal-weight adults: A randomized crossover trial. *Am J Clin Nutr*. 2013;98(2):358-366.
29. Liao Y, Basen-Engquist K, Urbauer DL, Bevers TB, Hawk E, Schembre SM. Using continuous glucose monitoring to motivate physical activity in overweight and obese adults: A pilot study. *Cancer Epidemiol Biomarkers Prev*. 2020;29(4):761-768.
30. Brannon G, Ray M, Cho P, et al. Utilizing biofeedback as a behavioral change strategy in overweight and obese cancer survivors to promote physical activity: A focus group analysis. In: Virtual; 2021.
31. O'Connor A. Can technology help us eat better? *The New York Times*. https://www.nytimes.com/2021/02/08/well/diet-glucose-monitor.html. Published February 8, 2021. Accessed March 8, 2021.
32. Hays RD, Bjorner JB, Revicki DA, Spritzer KL, Cella D. Development of physical and mental health summary scores from the patient-reported outcomes measurement information system (PROMIS) global items. *Qual Life Res*. 2009;18(7):873-880. doi:10.1007/s11136-009-9496-9
33. Morris NS, MacLean CD, Chew LD, Littenberg B. The Single Item Literacy Screener: evaluation of a brief instrument to identify limited reading ability. *BMC Fam Pract*. 2006;7(1):21.
34. Jeppesen KM, Coyle JD, Miser WF. Screening questions to predict limited health literacy: A cross-sectional study of patients with diabetes mellitus. *Ann Fam Med*. 2009;7(1):24-31.
35. Markland D, Tobin V. A modification to the behavioural regulation in exercise questionnaire to include an assessment of amotivation. *J Sport Exerc Psychol*. 2004;26(2):191-196.
36. Lerdal A, Moe B, Digre E, et al. Stages of Change–Continuous Measure (URICA-E2): psychometrics of a Norwegian version. *J Adv Nurs*. 2009;65(1):193-202.
37. Gellert P, Ziegelmann JP, Schwarzer R. Affective and health-related outcome expectancies for physical activity in older adults. *Psychol Health*. 2012;27(7):816-828.
38. Resnick B. Reliability and validity of the Outcome Expectations for Exercise Scale-2. *J Aging Phys Act*. 2005;13(4):382-394.
39. Kristal AR, Kolar AS, Fisher JL, et al. Evaluation of Web-Based, Self-Administered, Graphical Food Frequency Questionnaire. *J Acad Nutr Diet*. 2014;114(4):613-621. doi:10.1016/j.jand.2013.11.017

40. Pocock SJ, Simon R. Sequential treatment assignment with balancing for prognostic factors in the controlled clinical trial. *Biometrics*. 1975;103-115.
41. Pocock SJ. *Clinical Trials: A Practical Approach*. John Wiley & Sons; 2013.
42. Gibson B, Yingling L, Bednarchuk A, Janamatti A, Oakley-Girvan I, Allen N. An Interactive Simulation to Change Outcome Expectancies and Intentions in Adults With Type 2 Diabetes: Within-Subjects Experiment. *JMIR Diabetes*. 2018;3(1):e8069. doi:10.2196/diabetes.8069
43. American Diabetes Association. Standards of Medical Care in Diabetes—2017 Abridged for Primary Care Providers. *Clin Diabetes*. 2017;35(1):5-26. doi:10.2337/cd16-0067
44. Heikes KE, Eddy DM, Arondekar B, Schlessinger L. Diabetes Risk Calculator: A simple tool for detecting undiagnosed diabetes and pre-diabetes. *Diabetes Care*. 2008;31(5):1040-1045. doi:10.2337/dc07-1150
45. Warburton DER, Jamnik VK, Bredin SSD, Gledhill N. The Physical Activity Readiness Questionnaire for Everyone (PAR-Q+) and Electronic Physical Activity Readiness Medical Examination (ePARmed-X+). *Health Fit J Can*. 2011;4(2):3-17. doi:10.14288/hfjc.v4i2.103
